# Fluconazole Failure in the Treatment of Coccidioidal Meningitis

**DOI:** 10.3390/jof8111157

**Published:** 2022-11-01

**Authors:** Simran Gupta, Neil M. Ampel, Molly Klanderman, Marie F. Grill, Janis E. Blair

**Affiliations:** 1 Department of Internal Medicine, Mayo Clinic, Phoenix, AZ 85054, USA; 2 Division of Infectious Diseases, Mayo Clinic, Phoenix, AZ 85054, USA; 3 Department of Quantitative Health Sciences, Phoenix, AZ 85054, USA; 4 Department of Neurology, Mayo Clinic, Phoenix, AZ 85054, USA

**Keywords:** *Coccidioides*, coccidioidomycosis, meningitis, fungal meningitis, coccidioidal meningitis, fluconazole, antifungal treatment

## Abstract

Introduction: Coccidioidal meningitis (CM) is the most lethal form of disseminated coccidioidomycosis. Current guidelines recommend fluconazole as initial therapy but there has been a paucity of data regarding failure of fluconazole and optimal fluconazole dosage in the treatment of CM. We conducted this study to understand risk factors for fluconazole failure. Methods: We conducted a single-center retrospective chart review of patients diagnosed with coccidioidal meningitis between 1 January 1988 and 15 May 2021. Relevant demographic and clinical variables were collected, along with outcomes including treatment failure and death at any point. Univariate tests were conducted using the chi-squared goodness of fit test and analysis of variance. Results: Among 71 patients who began treatment for CM with fluconazole, 22 (31%) developed worsening meningitis at a median time of 206 days. Longer time from symptom onset to diagnosis of CM was a risk factor for fluconazole failure. Although the absolute failure rate of fluconazole starting dose of 400 mg daily was higher than that of 800 mg daily, the differences did not achieve statistical significance (*p* = 0.39). Conclusion: Fluconazole failure is not uncommon in the treatment of CM. A dose of 800 mg daily was not superior to a dose of 400 mg daily. All patients on fluconazole for CM require close monitoring.

## 1. Introduction

Coccidioidomycosis, caused by the dimorphic fungi *Coccidioides* spp., is endemic to portions of the western United States, northern Mexico, and parts of Central and South America. Inhalation of spores from the soil or air within the endemic area is the most common route of infection. While the precise number of coccidioidal infections is not known, the incidence appears to be rapidly increasing. Prior studies have shown that just in California, incidence increased by more than 213% (from 6.0/100,000 to 18.8/100,000) from 2014 to 2017 [1].

Disseminated infection outside the thoracic cavity occurs in approximately 1% to 5% of symptomatic persons [2]. Coccidioidal meningitis (CM) is the most lethal form of extrapulmonary infection and is the result of lymphohematogenous spread with subsequent granulomatous infection of the leptomeninges [3]. If untreated, CM has a high morbidity and mortality [4]. 

Therapy for CM changed dramatically in the 1990s when studies demonstrated that oral triazole antifungals were effective [5,6] and these agents soon replaced intrathecal amphotericin B as the treatment of choice. Fluconazole has been the accepted triazole antifungal for the treatment of CM based on oral bioavailability, CNS penetration, toxicity profile, and cost, compared to other agents [7]. Despite this, there has been a paucity of data regarding failure of fluconazole in the treatment of CM. In particular, there has been no clarity regarding optimal dosage, with some clinicians using 800 mg daily while others use 400 mg daily (or as much as 1200 mg). The purpose of this study was to understand the outcomes of patients with coccidioidal meningitis treated with fluconazole and, if possible, to identify risk factors for failure in such patients. 

## 2. Methods

We conducted a single-center retrospective chart review of patients from our institution with CM and identified potential patients using an electronic search of International Classification of Disease (ICD) versions 9 and 10, using codes 114.2, B38.4, and B38.9, respectively. We included all patients from 1 January 1988 to 15 May 2021 who were diagnosed with coccidioidal meningitis at Mayo Clinic in Arizona. We included patients with biochemical evidence of meningitis and one or more positive coccidioidal studies in the cerebrospinal fluid (CSF). We excluded patients without adequate treatment details, suspected but not laboratory-confirmed coccidioidal meningitis (i.e., no positive CSF serologic or other studies), those with disseminated coccidioidomycosis without meningitis, and those who were not initiated on fluconazole as the first line of treatment. This study was approved by the Mayo Clinic Institutional Review Board, and written informed consent was waived for those who provided research authorization.

From the medical record, we abstracted demographic characteristics, co-morbid illnesses, and duration of follow-up. We also tabulated the patient’s coccidioidal clinical symptoms at the time of diagnosis, cerebrospinal fluid (CSF) indices, including total and differential cell count, protein, and glucose, and CSF coccidioidal-specific tests such as serology (by enzyme immunoassay (EIA), immunodiffusion (ID), and complement fixation (CF)), antigen, fungal culture, and polymerase chain reaction (PCR), as well as details of chest and neurological imaging. We collected information on treatment, including specific medication, dosing, subsequent treatment regimens, order of treatment, and starting and ending doses of fluconazole.

CM was considered as present when there were abnormalities of CSF, including elevated nucleated cell count, elevated total protein, and decreased glucose, in conjunction with at least one of the following CSF abnormalities: (1) isolation of *Coccidioides* from CSF fungal culture; (2) positive *Coccidioides* PCR in the CSF; or (3) presence of serology by CF and/or ID for immunoglobulins IgM and IgG in CSF. Our primary study outcome was failure of fluconazole treatment. We defined fluconazole failure as any sustained increase in meningitis-related symptoms or progression of abnormalities of CSF and/or neuroimaging, with resultant medication change, either an increase in fluconazole dosage or change to another medication for reasons other than fluconazole adverse effects or drug toxicity. 

Descriptive statistics were used for data analysis. Demographics, clinical characteristics, treatment details, and outcomes were compared between patients with and without a history of prior pulmonary coccidioidomycosis. To identify predictors of fluconazole failure, demographics and clinical characteristics were also compared between patients who failed fluconazole and those who did not. Univariate tests were conducted using the chi-squared goodness of fit test and analysis of variance (ANOVA). A significance level of 0.05 was used. R statistical software version 4.0.3 was used to conduct the analysis. 

## 3. Results

From 1 January 1988 until 15 May 2021, we identified 102 subjects for possible inclusion. Thirty-one were excluded from further analysis; twenty-four were without CSF-specific microbiological evidence of CM, four had inadequate treatment details; and three were not started on fluconazole as initial therapy. Table 1 summarizes the respective demographic and clinical characteristics of the included 71 subjects. 

Among these 71 subjects, 22 (31.0%) experienced worsening meningitis (Table 2). Thirteen were receiving fluconazole 800 mg daily, eight were on 400 mg daily, and one was on 600 mg daily. Defining their fluconazole failure, all had increased symptoms, including headache, gait disturbances, stroke, and cognitive impairment. Among the 22, 11 had lumbar punctures documenting progressive inflammatory cerebrospinal fluid (CSF) findings; 14 had repeat imaging and all showed new or progressive abnormalities on imaging. Of all 71 patients in the study, 16 (22.5%) had fluconazole drug levels checked during treatment, and all 16 patients were therapeutic on their fluconazole. Of the 22 patients who failed fluconazole, 6 (27.3%) had fluconazole drug levels checked, and all 6 (100%) were therapeutic.

The median time to worsening meningitis was 206 days (range 30–7665). The median time to failure in those receiving fluconazole 800 mg daily was 313 days compared to 97 days for those on a lower dose (*p* = 0.24). In response to these failures, two subjects received an increased dosage of fluconazole and 19 received alternate triazole antifungal therapy. One was initiated on intrathecal amphotericin B. Ten patients (14.1%) ultimately died; only one died due to complications related to coccidioidal meningitis. 

We compared demographics, clinical characteristics, laboratory indices, and imaging findings between patients who had worsening meningitis and those who did not (Supplemental Table S1). Longer time from onset of initial neurologic symptoms (headache, confusion, dysarthria) to diagnosis of CM was found to be a significant predictor of fluconazole failure (301.7 days vs. 47.3 days, *p* = 0.03). The absence of encephalopathy on initial presentation was also found to be a predictor of fluconazole failure (4.5% vs. 28.6%, *p* = 0.03). The fluconazole starting dose was available for 70 subjects. Of the 17 subjects who started on 400 mg daily, seven (41.2%) experienced worsening meningitis, compared to 13 of the 44 subjects (29.5%) who started at 800 mg daily (*p* = 0.39).

We assessed whether the diagnosis and treatment of antecedent pulmonary coccidioidomycosis influenced the response to subsequent CM antifungal treatment. Nineteen of seventy-one (26.8%) patients were treated for pulmonary coccidioidomycosis prior to their CM. There were no significant demographic or laboratory differences between patients with or without prior coccidioidomycosis. All patients previously treated for pulmonary coccidioidomycosis were treated with fluconazole, and 40% were treated for their pulmonary coccidioidomycosis at a dose of 200 mg daily. On univariate logistic regression analysis, patients who were previously treated for pulmonary coccidioidomycosis were found to be 2.7 times more likely to fail fluconazole therapy (CI 0.9–8.22) than those who did not receive prior therapy.

We conducted a subgroup analysis excluding the 15 patients with hydrocephalus. As occurred in the entire group, absence of encephalopathy and longer time from symptom onset to diagnosis were found to be predictors of fluconazole failure (0% vs. 25%, *p* = 0.05; 404.6 days vs. 38.3 days, *p* = 0.01, respectively). In this subgroup analysis, increased CSF neutrophil count was found to be a predictor of fluconazole failure (39.5 vs. 18.8, *p* = 0.04). Patients who were previously treated for pulmonary coccidioidomycosis were more likely to fail fluconazole therapy (OR 3.40, CI 0.89–13.32).

## 4. Discussion

Prior to the advent of antifungal therapy, almost all cases of CM were fatal [4]. Intrathecal amphotericin was the first therapy for CM [8] and was used until oral triazole antifungals fluconazole and itraconazole were found to be effective [5,9]. In 1993, Galgiani and colleagues reported that subjects with coccidioidal meningitis who received 400 mg daily fluconazole had a response rate of 79%. Of ten who did not respond, six subsequently received 800 mg daily and four of these subjects responded. Thereafter, fluconazole became the standard of therapy due to its ease of administration and more favorable side effect profile when compared historically to intrathecal amphotericin [6]. Currently, guidelines from the IDSA recommend oral fluconazole at a dose of 400 to 1200 mg daily as initial therapy for CM [10]. Due to the high risk of recurrence following discontinuation of azole of treatment [11], it is recommended that patients receive lifelong therapy.

In the present study, we found that 22 subjects on fluconazole treated for CM developed worsening disease over time, for an overall failure rate of 31%. This is similar to a recently presented retrospective study that reported a fluconazole failure rate of 37% (n = 8/22) in children with coccidioidal meningitis in central California [12]. In the current study, there was a trend for fewer failures in those receiving 800 mg daily dosing of fluconazole compared to those receiving 400 mg daily dosing, but this was not significant. Of the 17 patients who had received 400 mg fluconazole daily, 7 (41.2%) failed vs. 13/44 (29.5%) who started at 800 mg (*p* = 0.39), with median intervals to failure being longer with the higher dose (-97 days vs. 313 days; *p* = 0.24). Johnson and colleagues noted a similar trend in their study of 34 subjects [13] in 1994. In addition, although the time to failure in those who ultimately failed was longer in those receiving 800 mg daily compared to those receiving less than this dose, this was not significantly different. Based on these studies, we cannot say that doses of fluconazole higher than 400 mg daily, as originally studied [6], result in fewer failures. While many clinicians prefer to use fluconazole at 800 mg daily, this must be tempered with the increased risk of adverse events and discontinuation associated with such higher doses [7].

We note that our study assessed a different endpoint (i.e., “fluconazole failure”), whereas the prospective Mycoses Study Group study assessed “fluconazole response,” defined as “40% or greater reduction in abnormalities without subsequent relapse during fluconazole treatment” [6]. It is difficult to reconcile these two definitions (i.e., fluconazole failure versus fluconazole response) because, in the current study, we were unable to systematically determine whether our patients had an initial response followed by subsequent failure. 

Another result of interest in this study was that 19/71 patients (26.8%) had received treatment for pulmonary coccidioidomycosis prior to the diagnosis of meningitis, with 8/19 receiving 200 mg fluconazole daily. The patients who were previously treated for pulmonary coccidioidomycosis, on univariate analysis, were 2.7 times (CI 0.9–8.22) more likely to fail therapy for meningitis. This finding is at odds with a recently published study which suggested that fluconazole can be used successfully for treatment of CM in patients with past outpatient fluconazole exposure (68% of patients responded to reinitiation of fluconazole) [14].

Additionally, we noted that subjects with a longer time to the diagnosis of CM were more likely to worsen on initial fluconazole therapy. These findings are congruent with prior observations that fewer patients respond to fluconazole if pretreatment symptoms included headache or meningismus compared with those who did not have such findings [6]. This observation underscores the importance of early recognition of CM by medical providers caring for patients reporting headaches and/or other neurologic symptoms, particularly in patients residing in or with travel to the *Coccidioides*-endemic area, and especially so in the context of known or prior coccidioidal pulmonary infection. A surprising finding was that the absence of encephalopathy on initial presentation appeared to be a predictor of worsening on fluconazole. We cannot explain this outcome but one possibility is that encephalopathic patients may have received a more expedited evaluation and earlier diagnosis of CM. Alternatively, patients without encephalopathy had fewer overall symptoms and may have been less adherent to their medication.

Following fluconazole failure for coccidioidal meningitis, the literature does not provide clear guidance. Itraconazole is a first-line alternative treatment [10], and studies support its efficacy in therapy [5,15]. However, poor absorption, need to monitor therapeutic drug levels, numerous drug–drug interactions, sodium absorption, and negative inotropic effects may limit its use [10]. In the present study, voriconazole was a common second-line treatment (15/22, 68.2%) though the need to maintain adequate therapeutic drug levels and adverse effects limited its long-term use. Posaconazole and isavuconazole have been helpful in case reports [9,16,17]. Liposomal amphotericin has been used as salvage monotherapy [18] or in combination with other azole treatments for the treatment of coccidioidal meningitis.

This study has several limitations, including a relatively small sample size, its observational nature and retrospective analysis, and a long inclusion period that does not account for changes in practice over time. Additionally, we note that a small percentage of our patient population was Hispanic/Latino, a group that is typically more common in southern Arizona, and therefore, we acknowledge that our population may not represent the general public, and thus our results may not be generalizable. Moreover, we did not systematically assess fluconazole adherence by therapeutic drug monitoring during the entire study period. Therapeutic drug monitoring of fluconazole has not generally been recommended given the >90% bioavailability of fluconazole, although some experts use such levels to assess patient medication adherence [19]. It is noteworthy that all fluconazole levels were therapeutic for the patients in the study when assessed. Finally, we rarely identified CM by culture, and therefore, antifungal susceptibility studies were not available.

In conclusion, we identified a high failure rate of initial fluconazole therapy for CM. These data suggest that close follow-up is imperative for all patients with CM, regardless of the dose of fluconazole.

## Figures and Tables

**Table 1:** The table displays the demographics and baseline characteristics of the 71 patients in this study.

Demographics and Characteristics of Coccidioidal Meningitis	Total (71)
Prior treatment of pulmonary coccidioidomycosis	19 (26.8%)
Mean time to follow-up (months)	85.4 (83.7)
Age at diagnosis	
Mean (SD)	55.1 (17.6)
Sex: Male	51 (71.8%)
Race	
White	57 (81.4%)
Asian	8 (11.4%)
Black or African American	3 (4.3%)
Native Hawaii/Pacific Islander	1 (1.4%)
Ethnicity	
Hispanic/Latino	5 (7.0%)
Not Hispanic/Latino	64 (90.1%)
Immunocompromised	
Yes	24 (34.3%)
Full time resident in endemic area	63 (88.7%)
Fluconazole as initial treatment for CM	71 (100%)
Starting dose of fluconazole (mg/d)	
200	2 (2.9%)
400	17 (24.3%)
600	6 (8.6%)
800	44 (62.9%)
1000 unknown	1 (1.4%) 1 (1.4%)

**Table 2:** Description of the 22 subjects who failed fluconazole therapy.

Patient	Fluconazole Dose (mg/day)	Time on Fluconazole (days)	Symptoms and Signs of Failure	Therapy Change
1	800	313	Episodic vomiting, increase in serum CF titer to 1:8, persistent CSF pleocytosis, and persistent extensive leptomeningeal enhancement on MRI	Voriconazole
2	800	7665	Progressive myelopathy with increased CSF CF to 1:8	Voriconazole
3	400	1695	Gait instability, central venous sinus thrombosis	Isavuconazole
4	600	30	Persistent headaches with CSF pleocytosis, increased protein with hypoglycorrhachia. Leptomeningeal enhancement and small vessel ischemic changes on MRI	Voriconazole
5	400	97	Gait changes and increased CSF cell count and protein with new leptomeningeal enhancement on MRI	Voriconazole
6	800	1545	Headaches; continued elevated serum CF titer of 1:128	Posaconazole
7	400	30	Right cerebrovascular accident	Fluconazole ↑800 mg/day
8	800	30	Worsening headache and dizziness	Voriconazole
9	800	150	Worsening headache and fatigue; CSF CF 1:32, new hydrocephalus with leptomeningeal enhancement on MRI	Voriconazole
10	800	30	Worsening headache; persistent CSF pleocytosis with irregular basilar enhancement on MRI	Voriconazole
11	800	685	Ataxia with headaches and memory loss; increasing CSF pleocytosis, protein with hypoglycorrhachia; new leptomeningeal enhancement and enlarging ventricles on MRI	Voriconazole
12	400	45	Increasing headaches and hydrocephalus	Voriconazole
13	800	1400	Persistent headaches and cognitive decline; CSF pleocytosis, elevated protein, hypoglycorrhachia and CSF CF 1:32, extensive leptomeningeal enhancement with enlarged ventricles on MRI	Posaconazole
14	400	2405	Pontine and basilar strokes; ataxia. Serum CF 1:16; brain abscess left pons with cisternal enhancement	Voriconazole
15	400	217	Persistent headache with intermittent vomiting. Possible seizure. CSF pleocytosis, elevated protein, hypoglycorrhachia with CSF CF 1:8; nodular, confluent leptomeningeal enhancement of basilar cisterns on MRI	Posaconazole
16	800	291	Cognitive impairment and lethargy	Itraconazole
17	800	194	Gait imbalance, cognitive decline. CSF pleocytosis with elevated protein and hypoglycorrhachia; spinal cord enhancement on MRI	Voriconazole
18	400	60	Increasing headache with lethargy	Fluconazole ↑600 mg/day
19	800	162	Weight loss, weakness, dizziness, difficulty eating	Voriconazole
20	400	166	Cognitive impairment, headache	Voriconazole
21	800	575	Headache, obstructed shunt	Voriconazole
22	800	379	Cognitive decline; hydrocephalus and diffuse leptomeningeal enhancement on MRI	Intrathecal amphotericin

Notes: CSF: cerebrospinal fluid; CF: complement-fixation antibody; MRI: magnetic resonance imaging.

## Data Availability

Due to privacy concerns, neither the data nor the source of the data can be made publicly available.

## Supplementary Materials

The following supporting information can be downloaded at: https://www.mdpi.com/article/10.3390/jof8111157/s1, Table S1 depicts all variables evaluated for effect on fluconazole failure with associated *p*-value and odds ratios. Data were analyzed using Pearson’s chi-squared test, linear model analysis of variance, and Fisher’s exact test. Risk factors for fluconazole failure in relation to initial dosage of fluconazole. Table S2 shows the distribution of the significant risk factors for failure of fluconazole (delay in time to initiation of therapy from onset of symptoms, and absence of encephalopathy on presentation) in the different dosage groups. Empty rows indicate missing data. One patient was initiated on fluconazole but the starting dosage was unknown.
