# Supplementary material for: Fluconazole Failure in the Treatment of Coccidioidal Meningitis
Source: J Fungi (Basel). 2022 Nov 1;8(11):1157. doi: 10.3390/jof8111157 (PMC9697806; doi:10.3390/jof8111157)
Supplement: Supplementary file 1 [file jof-08-01157-s001.zip › jof-2000049-supplementary.pdf]

Supplemental Table S1:

	Failure (N=22)	No failure (N=49)	Total (N=71)	P-value	Odds Ratio (CI)
Previous treatment of pulmonary coccidioidomycosis	9 (40.9%)	10 (20.4%)	19 (26.8%)	0.07[1]	2.7 (0.90-8.02)
Time from symptom onset to diagnosis of CM (days)	301.7 (641.3)	47.3 (52.4)	137.1 (395.8)	**0.03**[2]	1.01 (1.00-1.01)
Age at diagnosis	49.5 (18.0)	57.5 (17.0)	55.1 (17.6)	0.08[2]	0.97 (0.94-1.00)
Sex: Male	14 (63.6%)	37 (75.5%)	51 (71.8%)	0.30[1]	0.57 (0.19-1.72)
Race				0.32[3]	
White	16 (76.2%)	41 (83.7%)	57 (81.4%)		
Asian	2 (9.5%)	6 (12.2%)	8 (11.4%)		
Black Or African American	2 (9.5%)	1 (2.0%)	3 (4.3%)		
Native Hawaii/Pacific Islander	0 (0.0%)	1 (2.0%)	1 (1.4%)		
Other	1 (4.8%)	0 (0.0%)	1 (1.4%)		
Ethnicity				0.52[3]	
Hispanic/Latino	2 (9.1%)	3 (6.1%)	5 (7.0%)		
Not Hispanic/Latino	19 (86.4%)	45 (91.8%)	64 (90.1%)		
Choose Not To Disclose	1 (4.5%)	1 (2.0%)	2 (2.8%)		
Immunocompromised	6 (27.3%)	18 (37.5%)	24 (34.3%)	0.40[1]	0.63 (0.19-1.83)
Full-time resident in endemic area	20 (90.9%)	43 (87.8%)	63 (88.7%)	1.00[3]	
Cranial nerve findings	2 (9.1%)	6 (12.2%)	8 (11.3%)	1.00[3]	
Headache	20 (90.9%)	38 (77.6%)	58 (81.7%)	0.18[1]	2.89 (0.69-19.90)
Gait instability	7 (31.8%)	20 (40.8%)	27 (38.0%)	0.47[1]	0.68 (0.22-1.92)
Encephalopathy	1 (4.5%)	14 (28.6%)	15 (21.1%)	**0.03**[3]	
Fatigue	11 (50.0%)	17 (34.7%)	28 (39.4%)	0.22[1]	1.88 (0.68-5.30)
Fever/night sweats	11 (50.0%)	24 (49.0%)	35 (49.3%)	0.94[1]	1.04 (0.38-2.87)
Cough	8 (36.4%)	12 (24.5%)	20 (28.2%)	0.30[1]	1.76 (0.58-5.22)
Weight loss	4 (18.2%)	8 (16.3%)	12 (16.9%)	0.85[1]	1.14 (0.28-4.12)
Rash	4 (18.2%)	4 (8.2%)	8 (11.3%)	0.22[1]	2.50 (0.54-11.64)
Arthralgias	4 (18.2%)	5 (10.2%)	9 (12.7%)	0.35[1]	1.96 (0.44-8.23)
Serum WBC	8.5 (4.2)	8.2 (2.9)	8.3 (3.2)	0.78[2]	1.03 (0.83-1.28)
Serum eosinophils	0.7 (1.2)	1.9 (3.7)	1.6 (3.3)	0.32[2]	0.81 (0.39-1.10)
Serum IgM (immunodiffusion)	11 (50.0%)	16 (44.4%)	27 (46.6%)	0.68[1]	1.25 (0.43 – 3.66)
Serum IgG (immunodiffusion)	19 (86.4%)	30 (83.3%)	49 (84.5%)	0.76[1]	1.27 (0.30-6.57)
Serum IgM (EIA)	5 (26.3%)	16 (47.1%)	21 (39.6%)	0.14[1]	0.40 (0.11-1.31)
Serum IgG (EIA)	14 (73.7%)	26 (76.5%)	40 (75.5%)	0.82[1]	0.86 (0.24-3.31)
Serum Comp/Fix				0.35[3]	
1:2	3 (13.6%)	4 (11.4%)	7 (12.3%)		
1:4	6 (27.3%)	2 (5.7%)	8 (14.0%)		
1:8	0 (0.0%)	4 (11.4%)	4 (7.0%)		
1:16	6 (27.3%)	5 (14.3%)	11 (19.3%)		
1:32	2 (9.1%)	3 (8.6%)	5 (8.8%)		
1:64	1 (4.5%)	3 (8.6%)	4 (7.0%)		
1:128	2 (9.1%)	4 (11.4%)	6 (10.5%)		
1:256	1 (4.5%)	2 (5.7%)	3 (5.3%)		
1:512	0 (0.0%)	2 (5.7%)	2 (3.5%)		
1:2048	0 (0.0%)	1 (2.9%)	1 (1.8%)		
Negative	1 (4.5%)	5 (14.3%)	6 (10.5%)		
CSF RBC	762.2 (2738.2)	792.9 (2685.2)	784.0 (2675.2)	0.97[2]	1.00 (1.00-1.00)
CSF WBC	330.7 (433.2)	261.0 (392.4)	283.8 (403.9)	0.53[2]	1.00 (1.00-1.00)
CSF lymphocytes	52.8 (27.0)	63.5 (27.3)	59.8 (27.4)	0.17[2]	0.99 (0.96-1.01)
CSF monocytes	16.7 (16.0)	19.2 (22.6)	18.3 (20.3)	0.68[2]	0.99 (0.96-1.02)
CSF neutrophils	36.7 (29.2)	22.9 (25.8)	27.8 (27.6)	0.11[2]	1.02 (1.00-1.04)
CSF protein	297.1 (562.3)	146.6 (133.8)	196.0 (342.2)	0.11[2]	1.00 (1.00-1.01)
CSF glucose	45.3 (24.8)	48.9 (27.6)	47.8 (26.6)	0.63[2]	0.99 (0.97-1.02)
CSF IgM (immunodiffusion)	2 (10.5%)	1 (3.0%)	3 (5.8%)	0.55[3]	
CSF IgG (immunodiffusion)	9 (47.4%)	20 (60.6%)	29 (55.8%)	0.36[1]	0.59 (0.18 – 1.83)

CSF IgM (EIA)	5 (38.5%)	7 (28.0%)	12 (31.6%)	0.51[1]	1.61 (0.38-6.71)
CSF IgG (EIA)	11 (84.6%)	19 (76.0%)	30 (78.9%)	0.54[1]	1.74 (0.33-13.27)
CSF Comp/Fix				0.60[3]	
1:2	3 (18.8%)	6 (20.7%)	9 (20.0%)		
1:4	3 (18.8%)	7 (24.1%)	10 (22.2%)		
1:8	2 (12.5%)	3 (10.3%)	5 (11.1%)		
1:16	2 (12.5%)	8 (27.6%)	10 (22.2%)		
1:32	2 (12.5%)	1 (3.4%)	3 (6.7%)		
1:128	1 (6.2%)	0 (0.0%)	1 (2.2%)		
1:256	2 (12.5%)	1 (3.4%)	3 (6.7%)		
Negative	1 (6.2%)	3 (10.3%)	4 (8.9%)		
Any positive CSF serology	12 (92.3%)	21 (87.5%)	33 (89.2%)	1.00[3]	
Abnormal brain imaging	16 (84.2%)	25 (67.6%)	41 (73.2%)	0.18[1]	2.56 (0.58-12.52)
Imaging-Enhancement	14 (63.6%)	21 (42.9%)	35 (49.3%)	0.11[1]	2.33 (0.84-6.82)
Imaging-Stroke	4 (18.2%)	8 (16.3%)	12 (16.9%)	0.85[1]	1.14 (0.28-4.12)
Imaging-Hydrocephalus	6 (27.3%)	9 (18.4%)	15 (21.1%)	0.40[1]	1.67 (0.49-5.42)
Starting dose of Fluconazole (mg/d)				0.72[3]	
200	0 (0.0%)	2 (4.1%)	2 (2.9%)		
400	7 (33.3%)	10 (20.4%)	17 (24.3%)		
600	1 (4.8%)	5 (10.2%)	6 (8.6%)		
800	13 (61.9%)	31 (63.3%)	44 (62.9%)		
1000	0 (0.0%)	1 (2.0%)	1 (1.4%)		

Predictors of failure on Fluconazole. This supplemental table depicts all variables evaluated for effect on fluconazole failure with associated p-value and odds ratios. Data was analyzed using [1] Pearson's Chi-squared test, [2] Linear Model Analysis of Variance, [3] Fisher's Exact Test for Count Data. (abbreviations used: CSF = cerebrospinal fluid; EIA = enzyme immunoassay; IgM = Immunoglobulin M; IgG = Immunoglobulin G; Comp/Fix = Complement fixation; mg/d milligrams per day).

Supplemental Table S2:

Initial fluconazole Dosage	Fluconazole failure (yes/no)	Time from symptom onset to diagnosis(days)	Encephalopathy (yes/no)
200	no		no
200	no		no
400	yes	14	no
400	no		no
400	no	14	no
400	yes	6	yes
400	no	10	yes
400	yes		no
400	yes		no
400	no		yes
400	yes	14	no
400	no		no
400	no		no
400	no		no
400	no		no
400	yes	2232	no
400	no		no
400	no	30	no

400	yes	60	no
600	no		no
600	no		yes
600	no	65	no
600	no		no
600	no	4	no
600	yes	540	no
800	yes	16	no
800	no	9	yes
800	no	213	no
800	no	45	yes
800	no	31	no
800	yes		no
800	no	60	no
800	no	17	yes
800	no	20	yes
800	no	45	no
800	no	120	yes
800	no	10	no
800	no	25	yes
800	yes	20	no
800	no	31	no
800	no	15	no
800	yes	25	no
800	yes	120	no
800	yes	180	no
800	yes		no
800	no	7	no
800	yes	14	no
800	no	5	yes
800	no	210	yes
800	no	20	no
800	yes	60	no
800	yes	240	no
800	no	90	no
800	no	80	no
800	no	7	yes
800	no	50	no
800	no		no
800	no	15	no
800	no		no
800	no	120	no
800	no	14	no

800	no	45	yes
800	yes	45	no
800	no	60	no
initial dosage unknown	yes	30	no
800	yes	1800	no
800	no	25	yes
800	no		no
800	no		no
800	yes	15	no
1000	no	50	no

Risk factors for fluconazole failure in relation to initial dosage of fluconazole. Supplemental table S2 shows the distribution of the significant risk factors for failure of fluconazole (delay in time to initiation of therapy from onset of symptoms, and absence of encephalopathy on presentation) in the different dosage groups. Empty rows indicate missing data. One patient was initiated on fluconazole but the starting dosage was unknown.
